# Admission Day and Outcomes in Myocardial Infarction With Cardiogenic Shock: A Nationwide Propensity Score-Matched Study

**DOI:** 10.7759/cureus.100530

**Published:** 2025-12-31

**Authors:** Abdul Rasheed Bahar, George G Kidess, Yasemin Bahar, Matthew T Brennan, Denise Mourad, Ali Al-Ramadan, Olayiwola Bolaji, Mohammad Hazique, Wael AlJaroudi, M. Chadi Alraies

**Affiliations:** 1 Internal Medicine, Wayne State University, Detroit, USA; 2 Medicine, Wayne State University School of Medicine, Detroit, USA; 3 Medicine, Central Michigan University College of Medicine, Mount Pleasant, USA; 4 Internal Medicine, University of Maryland Capital Region Medical Center, Largo, USA; 5 Internal Medicine, Nuvance Health/Vassar Brothers Medical Center, Poughkeepsie, USA; 6 Cardiovascular Medicine, Wellstar Medical Colege of Georgia, Augusta, USA; 7 Cardiology, Detroit Medical Center, Detroit, USA

**Keywords:** nstemi, outcomes, stemi, weekdays, weekends

## Abstract

Background: The "weekend effect" refers to potential disparities in clinical outcomes based on the timing of hospital admission, with prior studies offering conflicting results, particularly in patients with acute myocardial infarction (AMI) complicated by cardiogenic shock (CS). This study evaluates the impact of admission timing on outcomes in patients with ST-elevation myocardial infarction and CS (STEMI-CS) and patients with non-STEMI and CS (NSTEMI-CS).

Methods: We conducted a retrospective analysis using the Nationwide Inpatient Sample from 2016 to 2021. Patients with STEMI-CS and NSTEMI-CS were identified using ICD-10 (International Classification of Diseases, Tenth Revision) codes and stratified by weekday versus weekend admissions. Propensity score matching and multivariable logistic regression were employed to adjust for confounders. The primary outcome was in-hospital mortality; secondary outcomes included acute stroke, pacemaker implantation, and resource utilization.

Results: Among 14,060 propensity-matched STEMI-CS patients (7,030 weekday and 7,030 weekend admissions), in-hospital mortality was higher in weekday compared to weekend admissions (2,373 (33.8%) vs. 2,261 (32.2%), p=0.044). Pacemaker implantation was less frequent in weekday admissions (28 (0.4%) vs. 55 (0.8%), p=0.003). Other outcomes, including percutaneous coronary intervention, acute stroke, sudden cardiac arrest, sepsis, pulmonary embolism, arrhythmias, and device implantation, were comparable between groups. Among 9,490 propensity-matched NSTEMI-CS patients (4,745 weekday and 4,745 weekend admissions), weekend admissions were associated with higher in-hospital mortality compared to weekdays (1,485 (31.3%) vs. 1,372 (28.9%), p=0.011), while other outcomes remained similar between groups.

Conclusion: Admission timing influences in-hospital mortality in patients with CS secondary to AMI, with contrasting trends observed between STEMI-CS and NSTEMI-CS cohorts. These findings suggest that variations in hospital protocols, staffing, or resource availability may contribute to these differences. Further research is warranted to elucidate the underlying mechanisms and develop strategies to optimize care delivery across all admission times.

## Introduction

Myocardial infarction (MI) is the most common cause of death worldwide and represents the most severe clinical manifestation of coronary artery disease (CAD), often associated with sudden cardiac death (SCD) [1]. Cardiovascular disease (CVD) was the underlying cause of 874,613 deaths in the United States in 2019, with a myocardial infarction occurring approximately every 40 seconds [2]. Timely intervention for patients with acute MI (AMI) is crucial to reducing morbidity and mortality [3]. Cardiogenic shock (CS) is a serious complication that can arise in these patients, requiring urgent management [4]. An ongoing debate explores whether hospital admission on weekdays versus weekends influences timeliness and quality of care. Weekend admissions are often associated with reduced staffing levels and limited on-site availability of essential specialists [5], which may result in delays in diagnosis and treatment, including percutaneous coronary intervention (PCI) and mechanical circulatory support.

The phenomenon known as the “weekend effect” has been highlighted in previous studies, suggesting improved outcomes and lower mortality rates for weekday admissions, likely due to greater availability of personnel and resources [6]. This effect is particularly pronounced in ST-elevation myocardial infarction (STEMI) patients, who require immediate revascularization and shorter door-to-balloon times [7]. However, other studies have found no significant differences in outcomes between weekday and weekend admissions for AMI patients [8-10].

In both STEMI and non-STEMI (NSTEMI) patients where the condition is complicated by cardiogenic shock, prompt revascularization is required to improve survival [10]. These patients are often admitted to or transferred to larger centers with dedicated cardiac critical care units, where specialized shock teams are expected to be available [11]. However, there is limited data examining outcomes in this specific subset of patients. The goal of our study was to evaluate the impact of the day of admission on cardiovascular outcomes in STEMI-CS and NSTEMI-CS patients.

## Materials and methods

Data source

We utilized the Nationwide Inpatient Sample (NIS) data set from 2016 to 2021 to identify patients with a principal diagnosis of STEMI and NSTEMI with concomitant cardiogenic shock. NIS, managed by the Agency for Healthcare Research and Quality (AHRQ) and Healthcare Cost and Utilization Project (HCUP), is the largest publicly available all-payer inpatient healthcare database in the United States, providing nationally representative estimates of inpatient utilization, access, outcomes, cost, and quality [12]. The NIS data set covers 97% of total U.S. hospitalizations from 48 states, including Michigan, participating in HCUP. A 20% random sample of patients within each stratum is selected, and their demographic information, diagnoses, and resource utilization are entered into the database. Each discharge is then weighted (weight = total number of discharges from all U.S. acute care hospitals/number of discharges in the 20% sample) to ensure the NIS is nationally representative. It contains a primary discharge diagnosis and can have up to 39 secondary diagnoses, including comorbid conditions and complications that occur during hospitalization. The database also provides information regarding patient demographic information, resource utilization such as length of stay (LOS) and total hospitalization charges, hospital characteristics, insurance status, in-hospital outcomes, and primary and secondary procedures, all identified using codes from the International Classification of Diseases, Ninth/Tenth Revision, Clinical Modification (ICD-9/10-CM) [12]. Our study follows the methodological design checklist proposed by Khera et al. to address common study design errors in NIS-based research [13]. Since the NIS is a publicly available, anonymized national database with prior ethical approval from the AHRQ, our study was exempt from Institutional Review Board (IRB) approval.

Study population

Patients were identified using ICD-10 codes as follows: STEMI was defined by codes I21.0, I21.1, I21.2, I21.3, and I21.4; NSTEMI by code I21.4; and cardiogenic shock by code R57.0. Patients were excluded from the analysis if they were younger than 18 years old. Subsequently, patients were stratified into two cohorts according to their day of admission: weekend admissions and weekday admissions.

Definitions and outcomes

The primary outcome was in-hospital all-cause mortality. Secondary outcomes were as follows: (i) morbidity measured by the development of acute heart failure, sudden cardiac arrest (SCA), acute stroke, need for percutaneous coronary intervention (PCI), mechanical circulatory support (MCS) utilization, need for pacemaker insertion and defibrillation, pulmonary embolism (PE), sepsis, and cardiac arrhythmias and (ii) resource utilization measured by LOS and total hospitalization charges and costs. All the abovementioned outcomes were identified using ICD-10-CM diagnoses and procedure codes (Supplementary materials 1-3). Patients’ demographics, LOS, and total hospitalization charges were directly obtained from the NIS database. Total hospital charges reflect the amount a patient was billed for the entire hospital stay, but they do not reflect the actual cost of care. The HCUP provides data with hospital-specific cost-to-charge ratios based on inpatient costs across all payers [14]. Using this information, total hospital costs were calculated by multiplying the hospital charges by the corresponding cost-to-charge ratio. All hospitalization costs and charges were adjusted for inflation over time using the hospital services category of the consumer price index and are represented in 2021 US dollars [15].

Statistical analysis

All statistical analyses were performed using STATA statistical software, version 18 (Stata Corp LLC, College Station, TX), with a two-sided p-value <0.05 considered statistically significant. Weighted sampling analyses were utilized to accurately estimate the national average, in compliance with the HCUP guidelines [16]. Categorical variables were compared using the chi-square test for unadjusted analyses and are presented as frequencies and percentages. Continuous variables were compared using the Student’s t-test in the unadjusted analyses and are presented as means ± SDs. Continuous variables were tested for normality of distribution using the Shapiro-Wilk test and reported as means with SD or medians with interquartile ranges (IQRs), depending on their distribution. Missing data were not excluded from the analysis; instead, we addressed and treated the missing data using multiple imputations by predictive mean matching, preserving the distribution and variability of the observed data while incorporating the relationships among variables (Supplementary material 4) [17,18].

A two-staged multivariable mixed-effects logistic regression analysis was employed to identify independent predictors of study outcomes and in-hospital complications. The analysis incorporated patient-level variables such as age, sex, race, medical comorbidities, and socioeconomic status, as well as hospital-level variables such as size, type, location, nature, and day of admission (Supplementary material 5). To further mitigate baseline population discrepancies, we utilized propensity score matching. Propensity score matching was performed using 1:1 nearest-neighbor matching without replacement and a caliper width of 0.2 of the standard deviation of the logit of the propensity score. Covariate balance was assessed using standardized mean differences, with adequate balance prespecified as an absolute standardized mean difference <0.10 for all variables [19].

## Results

Patient and hospital characteristics

**Figure 1:**
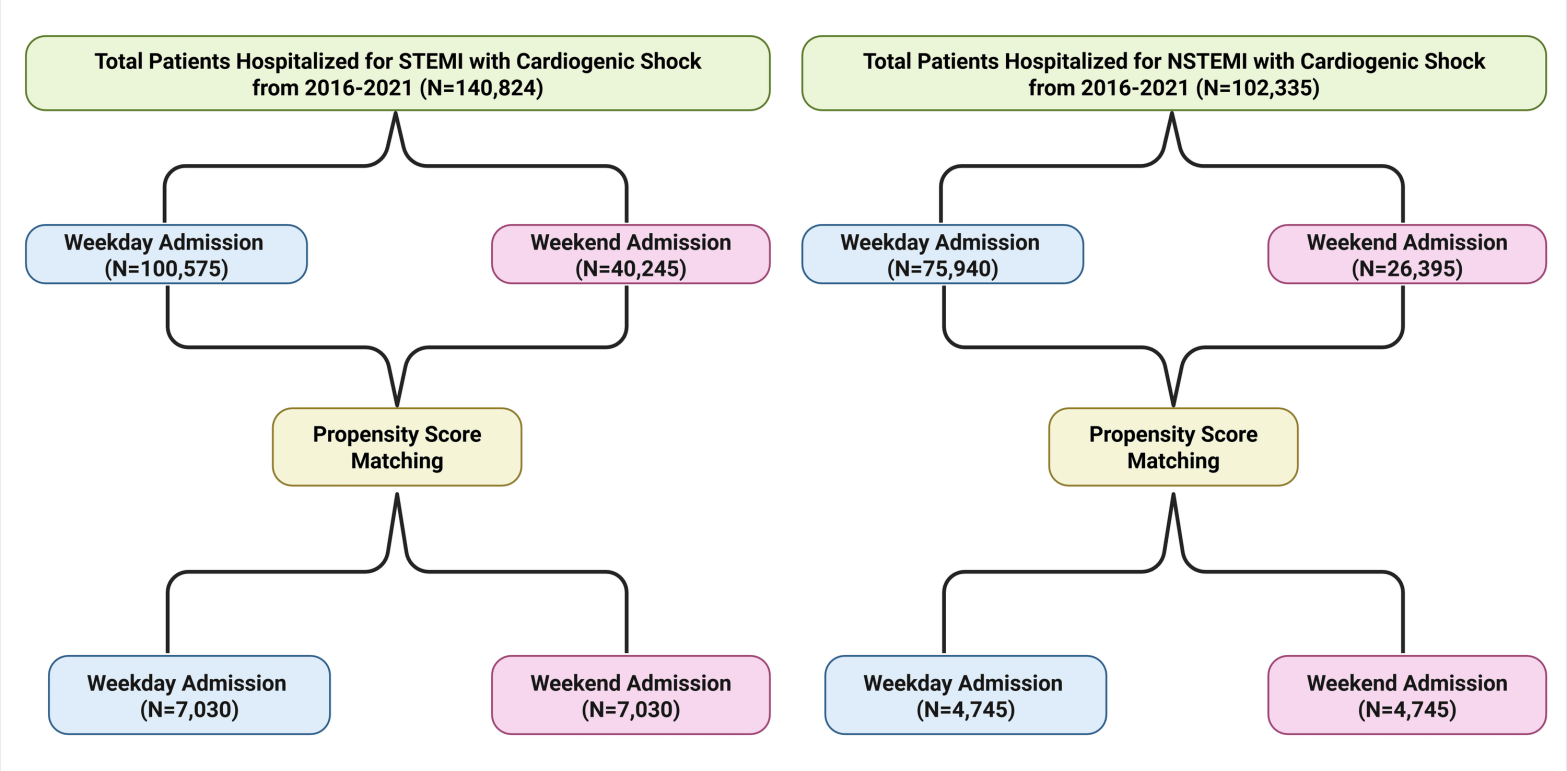
Flow diagram of the included study population

A total of 140,824 patients with STEMI-CS were identified, of whom 100,575 (71.4%) were admitted on weekdays and 40,245 (28.6%) on weekends. Additionally, 102,335 patients with NSTEMI-CS were identified, including 75,940 (74.2%) weekday admissions and 26,395 (25.8%) weekend admissions (Figure 1). Among patients with STEMI-CS, the mean age was 66.7 years for weekday admissions and 66.3 years for weekend admissions. In the NSTEMI-CS cohort, the mean age was 70.2 years for weekday admissions and 70.6 years for weekend admissions (Supplementary materials 6-7). As shown in Table 1, 66.4% of patients with STEMI-CS admitted on weekdays were male, compared with 67.8% of those admitted on weekends.

**Table 1:** Baseline characteristics of patients with acute MI with CS based on admission day Categorical variables were compared using chi-square tests and are presented as n (%). Continuous variables were assessed with the Shapiro–Wilk test for normality and compared using Student’s t-test or Wilcoxon rank-sum test, as appropriate, and are presented as means ± SDs or median (IQR). STEMI-CS: ST-elevation myocardial infarction and cardiogenic shock; NSTEMI-CS: non-STEMI and CS; MI: myocardial infarction; PCI: percutaneous coronary intervention; CABG: coronary artery bypass graft; OSA: obstructive sleep apnea

Baseline characteristics	STEMI-CS (N = 140,824)				NSTEMI-CS (N = 102,335)			
	Weekday admission (Mon-Fri)	Weekend admission (Sat/Sun)	p-value	Test statistic	Weekday admission (Mon-Fri)	Weekend admission (Sat/Sun)	p-value	Test statistic
	n = 100,575	n = 40,245			n = 75,940	n = 26,395		
	n (%)	n (%)			n (%)	n (%)		
Mean age (standard deviation)	66.69 (12.45)	66.31 (12.61)	0.092	1.35	70.17 (11.75)	70.56 (11.59)	0.075	1.23
Indicator of sex (%)								
Male	66,775 (66.4)	27,270 (67.8)	0.027	4.92	48,065 (64.1)	16,555 (62.7)	0.063	3.46
Female	33,780 (33.6)	12,970 (32.2)			26,870 (35.9)	9,840 (37.3)		
Year (%)								
2016	16,160 (16.1)	6,680 (16.6)	0.266	1.29	12,000 (16)	4,360 (16.5)	0.330	1.15
2017	16,290 (16.2)	6,710 (16.7)			12,525 (16.7)	4,515 (17.1)		
2018	17,090 (17)	6,560 (16.3)			12,640 (16.9)	4,530 (17.2)		
2019	17,705 (17.6)	6,740 (16.7)			13,200 (17.6)	4,530 (17.2)		
2020	16,375 (16.3)	6,895 (16.5)			12,055 (16.1)	3,920 (14.9)		
2021	16,955 (16.9)	6,895 (17.1)			12,520 (16.7)	4,540 (17.2)		
Race (%)								
White	70,175 (76.8)	28,210 (76.9)	0.291	1.24	51,855 (74.5)	17,985 (73.3)	0.348	1.11
Black	7,590 (8.3)	3,215 (8.8)			7,090 (10.2)	2,655 (10.8)		
Hispanic	9,170 (10)	3,425 (9.3)			7,210 (10.3)	2,610 (10.6)		
Asian/Pacific Islander	3,880 (4.2)	1,550 (4.2)			2,930 (4.2)	1,020 (4.2)		
Native American	580 (0.6)	275 (0.7)			555 (0.8)	250 (1)		
Insurance type (%)								
Medicare	54,195 (55.7)	21,345 (54.7)	0.047	2.42	50,965 (70.2)	18,220 (70.9)	0.325	1.16
Medicaid	10,525 (10.8)	4,110 (10.5)			5,925 (8.2)	2,215 (8.6)		
Private insurance	26,335 (27)	11,075 (28.4)			13,220 (18.2)	4,425 (17.2)		
Self-pay	5,955 (6.1)	2,290 (5.9)			2,345 (3.2)	810 (3.2)		
No charge	365 (0.4)	215 (0.6)			155 (0.2)	35 (0.1)		
Type of admission (%)								
Non-elective	96,775 (96.4)	39,015 (97.1)	0.004	8.47	70,880 (94.7)	25,590 (97.1)	<0.001	50.83
Elective	3,615 (3.6)	1,160 (2.9)			3,935 (5.3)	765 (2.9)		
Hospital characteristic: hospital bed size (%)								
Small	14,340 (14.3)	5,585 (13.9)	0.30	1.2	10,765 (14.4)	3,825 (14.5)	0.973	0.03
Medium	28,630 (28.5)	11,200 (27.8)			20,780 (27.7)	7,295 (27.6)		
Large	57,605 (57.3)	23,460 (58.3)			43,395 (57.9)	15,275 (57.9)		
Hospital characteristic: teaching status (%)								
Rural	4,595 (4.6)	1,995 (5)	0.273	1.3	3,330 (4.4)	1,165 (4.4)	0.025	3.7
Urban non-teaching	19,275 (19.2)	7,520 (18.7)			12,495 (16.7)	4,830 (18.3)		
Urban teaching	76,705 (76.3)	30,730 (76.4)			59,115 (78.9)	20,400 (77.3)		
Hospital region (%)								
Northeast	16,335 (16.2)	6,575 (16.3)	0.044	2.7	12,245 (16.3)	3,890 (14.7)	0.004	4.49
Midwest	22,020 (21.9)	9,380 (23.3)			16,110 (21.5)	5,505 (20.9)		
South	39,940 (39.7)	15,760 (39.2)			30,780 (41.1)	10,930 (41.4)		
West	22,280 (22.2)	8,530 (21.2)			15,805 (21.1)	6,070 (23)		
Transfer-in status (%)								
Not transferred	75,265 (75.6)	30,235 (75.9)	0.235	1.44	49,390 (66.3)	18,045 (68.7)	0.007	5.02
Transferred	21,575 (21.7)	8,380 (21)			22,330 (30)	7,270 (27.7)		
Transfer-out indicator (%)								
Not transferred	76,465 (76.1)	30,850 (76.7)	0.483	0.73	50,765 (67.8)	17,605 (66.7)	0.029	3.53
Transferred to a different acute care hospital	8,055 (8)	3,095 (7.7)			5,920 (7.9)	2,390 (9.1)		
Transferred to a different type of health facility	16,015 (15.9)	6,270 (15.6)			18,230 (24.3)	6,385 (24.2)		
Comorbidities (%)								
Protein energy malnutrition	5,715 (5.7)	2,055 (5.1)	0.051	3.82	6,745 (9)	2,355 (8.9)	0.863	0.03
Obesity	17,035 (16.9)	6,900 (17.1)	0.678	0.17	15,075 (20.1)	5,255 (19.9)	0.748	0.10
Hypertension	70,810 (70.4)	27,955 (69.5)	0.120	2.42	62,405 (83.3)	21,895 (83)	0.597	0.28
Dyslipidemia	51,235 (50.9)	20,415 (50.7)	0.740	0.11	42,600 (56.8)	15,005 (56.8)	0.998	<0.001
Smoker	23,115 (23)	9,050 (22.5)	0.371	0.80	18,505 (24.7)	6,160 (23.3)	0.048	3.90
Prior MI	10,990 (10.9)	4,060 (10.1)	0.040	4.23	11,495 (15.3)	4,090 (15.5)	0.786	0.07
Prior PCI	10,730 (10.7)	4,080 (10.1)	0.182	1.78	9,290 (12.4)	3,370 (12.8)	0.485	0.49
Prior CABG	3,965 (3.9)	1,460 (3.6)	0.214	1.55	6,975 (9.3)	2,545 (9.6)	0.472	0.52
OSA	5,330 (5.3)	2,070 (5.1)	0.591	0.29	6,195 (8.3)	2,280 (8.6)	0.400	0.71
Pulmonary disease	13,995 (13.9)	5,370 (13.3)	0.214	1.55	15,325 (20.4)	5,350 (20.3)	0.774	0.08
Immunocompromised	90 (0.1)	30 (0.1)	0.702	0.15	45 (0.1)	25 (0.1)	0.409	0.68
Hypothyroidism	9,380 (9.3)	3,330 (8.3)	0.006	7.69	9,340 (12.5)	3,295 (12.5)	0.970	0.001
Anemia	3,730 (3.7)	1,365 (3.4)	0.205	1.61	4,150 (5.5)	1,565 (5.9)	0.292	1.11
Diabetes mellitus	34,140 (33.9)	13,520 (33.6)	0.575	0.31	35,050 (46.8)	12,555 (47.6)	0.328	0.96
Liver disease	1,985 (2)	905 (2.2)	0.146	2.11	2,145 (2.9)	780 (3)	0.729	0.12

A total of 64.1% of patients with NSTEMI-CS admitted on the weekday were male patients, while 62.7% of NSTEMI-CS patients admitted on the weekend were male patients. The majority of patients were White in both disease groups, regardless of the day of admission. With regard to comorbidities, patients with STEMI-CS admitted on the weekday had a higher prevalence of prior myocardial infarction (10.9% vs. 10.1%, p=0.040) and a higher prevalence of hypothyroidism (9.3% vs. 8.3%, p=0.006). For patients admitted with NSTEMI-CS, the only significantly different comorbidity was a higher prevalence of smoking in patients admitted on the weekday (24.7% vs. 23.3%, p=0.048).

Crude and adjusted outcomes

On unadjusted analysis (Table 2), patients with STEMI-CS admitted on the weekday had significantly lower odds of acute stroke compared to those admitted on the weekend (OR: 0.70, CI: 0.56-0.87, p=0.001).

**Table 2:** Univariable logistic regression analysis of outcomes in acute MI patients with CS based on admission day OR: unadjusted odds ratio; CI: confidence interval; STEMI-CS: ST-elevation myocardial infarction and cardiogenic shock; NSTEMI-CS: non-STEMI and CS; PCI: percutaneous coronary intervention; SCA: sudden cardiac arrest; PE: pulmonary embolism

Outcomes	STEMI-CS		NSTEMI-CS	
	OR (95% CI)	p-value	OR (95% CI)	p-value
In-hospital mortality	0.96 (0.91-1.02)	0.160	1.10 (1.02-1.17)	0.009
PCI	1.012 (0.96-1.06)	0.651	1.03 (0.94-1.12)	0.559
Acute stroke	0.70 (0.56-0.87)	0.001	1.19 (0.92-1.54)	0.182
SCA	0.98 (0.92-1.05)	0.635	0.98 (0.90-1.07)	0.679
PE	1.02 (0.79-1.32)	0.857	0.84 (0.63-1.11)	0.219
Sepsis	0.94 (0.84-1.04)	0.238	1.01 (0.91-1.12)	0.858
Functional decline	0.56 (0.12-2.58)	0.452	0.95 (0.19-4.69)	0.946
Cardiac arrhythmias	1.01 (0.96-1.07)	0.712	1.00 (0.94-1.07)	0.898
Pacemaker implantation	091 (0.68-1.22)	0.537	1.11 (0.85-1.44)	0.448
Defibrillator implantation	0.91 (0.734-1.12)	0.368	0.85 (0.77-1.17)	0.612

There were no statistically significant differences in other unadjusted outcomes for patients with STEMI-CS based on the day of admission, including in-hospital mortality, PCI, SCA, pulmonary embolism, sepsis, functional decline, cardiac arrhythmias, pacemaker implantation, or defibrillator implantation (p>0.05). Patients with NSTEMI-CS admitted on the weekday were found to have higher odds of in-hospital mortality compared to patients admitted on the weekend (OR: 1.10, CI: 1.02-1.17, p=0.009). There were no statistically significant differences in other unadjusted outcomes for patients with NSTEMI-CS based on the day of admission, including percutaneous coronary intervention, acute stroke, sudden cardiac arrest, pulmonary embolism, sepsis, functional decline, cardiac arrhythmias, pacemaker implantation, or defibrillator implantation (p>0.05).

After multivariate logistic regression analysis (Table 3), patients with STEMI-CS admitted on the weekday continued to show significantly lower odds of acute stroke compared to those admitted on the weekend (adjusted OR, or aOR: 0.75, CI: 0.60-0.96, p=0.016).

**Table 3:** Multivariable logistic regression analysis of outcomes in patients with acute MI with CS based on admission day aOR: adjusted odds ratio; CI: confidence interval; STEMI-CS: ST-elevation myocardial infarction and cardiogenic shock; NSTEMI-CS: non-STEMI and CS; PCI: percutaneous coronary intervention; SCA: sudden cardiac arrest; PE: pulmonary embolism

Outcomes	STEMI-CS		NSTEMI-CS	
	aOR (95% CI)	p-value	aOR (95% CI)	p-value
In-hospital mortality	0.97 (0.91-1.04)	0.407	1.15 (1.05-1.25)	<0.001
PCI	1.00 (0.95-1.06)	0.950	1.02 (0.93-1.12)	0.606
Acute stroke	0.75 (0.60-0.96)	0.016	1.11 (0.84-1.47)	0.446
SCA	0.96 (0.90-1.03)	0.287	0.97 (0.89-1.06)	0.526
PE	1.10 (0.84-1.44)	0.498	0.79 (0.59-1.06)	0.115
Sepsis	0.95 (0.85-1.05)	0.282	1.01 (0.91-1.13)	0.845
Functional decline	0.17 (0.019-1.49)	0.110	0.97 (0.18-5.21)	0.971
Cardiac arrhythmias	0.98 (0.93-1.04)	0.615	0.99 (0.93-1.06)	0.858
Pacemaker implantation	0.96 (0.70-1.30)	0.783	1.08 (0.82-1.42)	0.595
Defibrillator implantation	0.95 (0.76-1.19)	0.677	0.94 (0.75-1.18)	0.597

There were no statistically significant differences in other unadjusted outcomes for patients with STEMI-CS based on the day of admission, including in-hospital mortality, PCI, SCA, PE, sepsis, functional decline, cardiac arrhythmias, pacemaker implantation, or defibrillator implantation (p>0.05). Patients with NSTEMI-CS admitted on the weekday were also found to have higher odds of in-hospital mortality compared to patients admitted on the weekend (aOR: 1.15, CI: 1.05-1.25, p<0.001). There were no statistically significant differences in other unadjusted outcomes for patients with NSTEMI-CS based on the day of admission, including percutaneous coronary intervention, acute stroke, sudden cardiac arrest, pulmonary embolism, sepsis, functional decline, cardiac arrhythmias, pacemaker implantation, or defibrillator implantation (p>0.05).

Propensity score-matched outcomes

**Figure 2:**
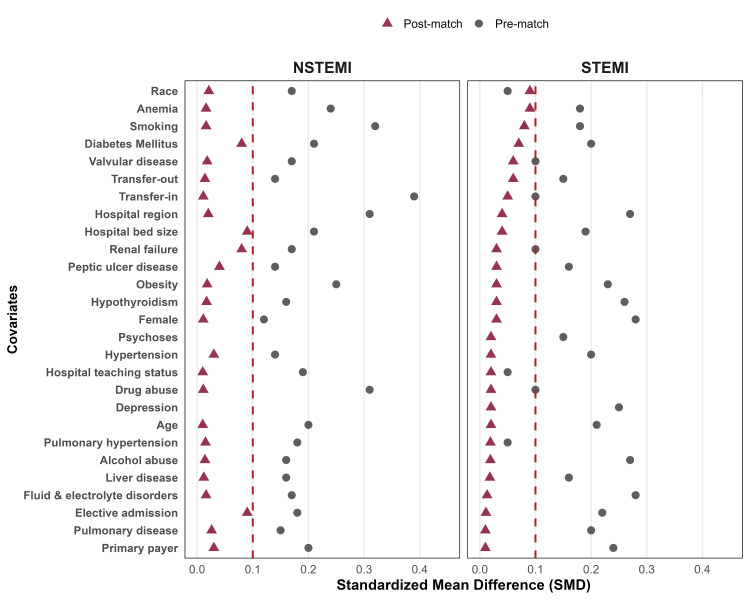
Covariate balance before and after propensity score matching in STEMI and NSTEMI cohorts SMDs for baseline covariates are shown before (circles) and after (triangles) propensity score matching for patients with NSTEMI (left panel) and STEMI (right panel). The vertical dashed line at SMD = 0.10 represents the prespecified threshold for adequate covariate balance. Post-matching SMDs <0.10 across covariates indicate a successful balance between weekday and weekend admission groups. STEMI: ST-elevation myocardial infarction; NSTEMI: non-STEMI

**Figure 3:**
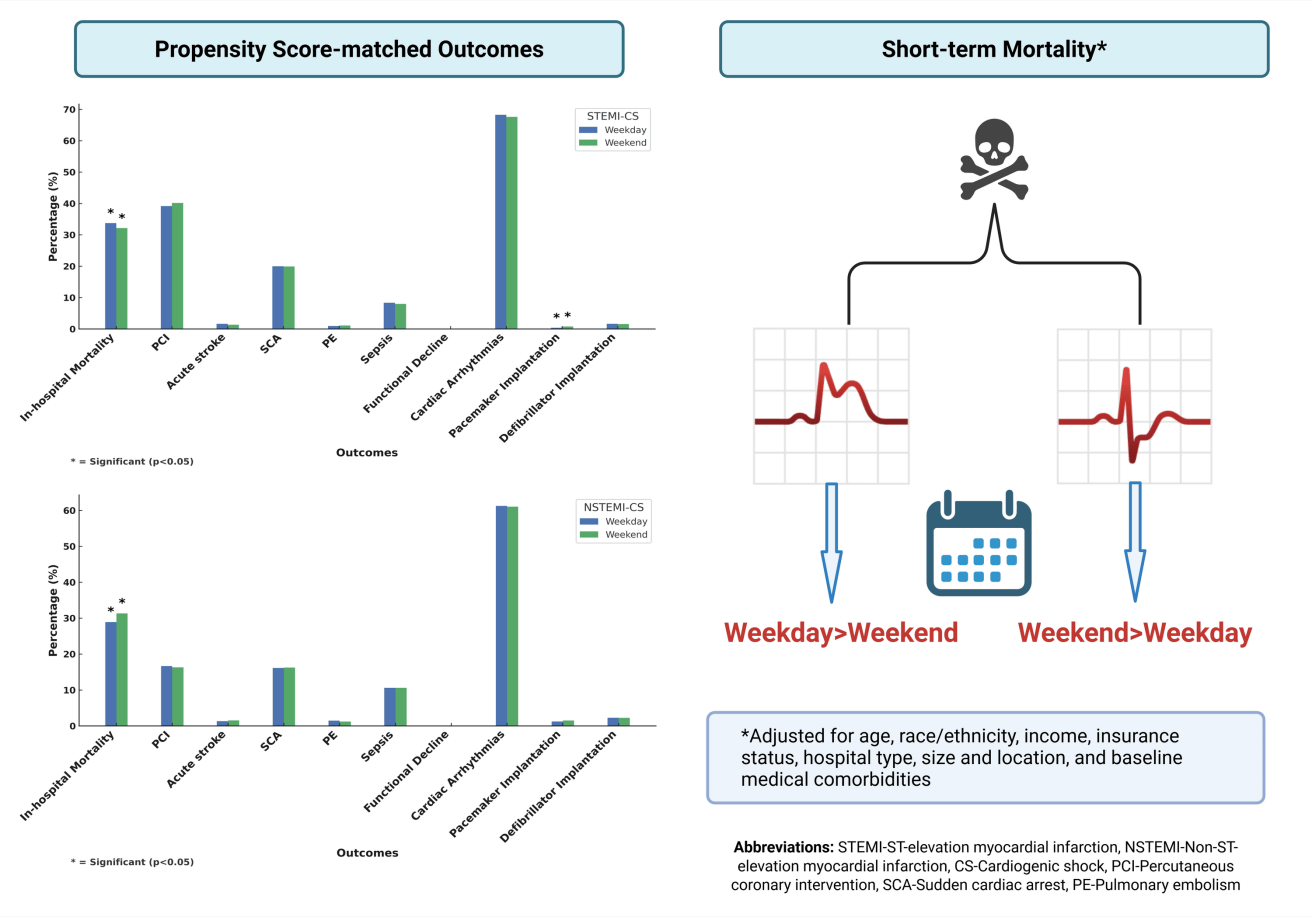
Impact of admission day on outcomes in patients with acute myocardial infarction with cardiogenic shock Created with BioRender.com (BioRender, Toronto)

To further account for confounding factors, we performed propensity score matching (Figure 2; Supplementary materials 8-9). For patients with STEMI-CS, we found a significantly higher incidence of in-hospital mortality for those admitted during the weekday compared to the weekend (33.76% vs. 32.16%, p=0.044), as well as a significantly lower incidence of pacemaker implantation for patients admitted on the weekday (0.40% vs. 0.78%, p=0.003). We found no further difference in other outcomes for the STEMI-CS group based on the day of admission, including PCI, sudden cardiac arrest, pulmonary embolism, sepsis, functional decline, cardiac arrhythmias, pacemaker implantation, or defibrillator implantation (p>0.05). For patients with NSTEMI-CS, we found a significantly higher in-hospital mortality for patients admitted on the weekend compared to the weekday (28.91% vs. 31.30%, p=0.011). There were no statistically significant differences in other outcomes for patients with NSTEMI-CS based on the day of admission, including PCI, acute stroke, SCA, PE, sepsis, functional decline, cardiac arrhythmia, pacemaker implantation, or defibrillator implantation (p>0.05) (Table 4). Figure 3 shows the propensity score-matched outcomes for STEMI-CS and NSTEMI-CS patients by weekday and weekend admissions.

**Table 4:** Propensity-matched outcomes in patients with acute MI with cardiogenic shock based on admission day After propensity score matching, outcomes were compared between weekend and weekday admissions using logistic regression and Pearson’s chi-square test. STEMI-CS: ST-elevation myocardial infarction and cardiogenic shock; NSTEMI-CS: non-STEMI and CS; PCI: percutaneous coronary intervention; SCA: sudden cardiac arrest; PE: pulmonary embolism

Outcomes	STEMI-CS				NSTEMI-CS			
	Weekday (N=7,030)	Weekend (N=7,030)	p-value	Pearson’s χ²	Weekday (N=4,745)	Weekend (N=4,745)	p-value	Pearson’s χ²
	n (%)	n (%)			n (%)	n (%)		
In-hospital mortality	2,373 (33.76)	2,261 (32.16)	0.044	4.03	1,372 (28.91)	1,485 (31.30)	0.011	6.39
PCI	2,753 (39.16)	2,824 (40.17)	0.221	1.50	790 (16.65)	773 (16.29)	0.638	0.22
Acute stroke	116 (1.65)	95 (1.35)	0.145	2.12	62 (1.31)	73 (1.54)	0.340	0.90
SCA	1,405 (19.99)	1,400 (19.91)	0.916	0.01	765 (16.12)	771 (16.25)	0.867	0.02
PE	66 (0.94)	78 (1.11)	0.315	1.01	70 (1.48)	57 (1.2)	0.245	1.34
Sepsis	587 (8.35)	562 (7.99)	0.442	0.59	504 (10.62)	503 (10.6)	0.973	0.00
Functional decline	5 (0.07)	1 (0.01)	0.102	2.66	2 (0.04)	2 (0.04)	1.000	0.00
Cardiac arrhythmias	4,799 (68.26)	4,754 (67.62)	0.416	0.66	2,906 (61.24)	2,896 (61.03)	0.833	0.04
Pacemaker implantation	28 (0.40)	55 (0.78)	0.003	8.83	58 (1.22)	71 (1.5)	0.249	1.32
Defibrillator implantation	116 (1.65)	111 (1.58)	0.738	0.11	107 (2.26)	106 (2.23)	0.945	0.01
Mean length of stay (days)	7.66	7.52	0.245	-	10.48	10.64	0.165	-
Mean hospital charges (USD)	240,384.1	227,515.7	0.092	-	264,218	260,811.7	0.084	-

Resource utilization

With regard to healthcare resource utilization, the mean LOS was similar based on the day of admission for both the STEMI-CS and NSTEMI-CS groups (less than one day difference for both patient populations). Mean hospital charges were higher for weekday admissions for both disease groups (STEMI-CS, $240,384.1 vs $227,515.7; NSTEMI-CS, $264,218 vs. $260,811.7) (Table 4). Most hospital admissions for both groups were in urban, teaching hospital settings and large hospitals. The majority of patients were admitted to hospitals in the Southern region in both the STEMI-CS (39.7% on the weekday, 39.2% on the weekend) and NSTEMI-CS (41.1% on the weekday, 41.4% on the weekend) groups. The majority of patients in this study utilized Medicare as their primary insurance regardless of their disease type or day of admission (Table 1).

## Discussion

Cardiogenic shock, in both STEMI and NSTEMI patients, requires prompt revascularization for effective management [11]. Studies have found a “weekend effect” when it comes to the management and clinical outcomes of patients experiencing STEMI or NSTEMI, with higher mortality rates seen on weekend admissions [20, 21]. However, other studies have not been able to reproduce this effect [8,9].

Our study provides key insights into the “weekend effect” for patients with STEMI-CS and NSTEMI-CS, particularly regarding in-hospital mortality and comorbidities. First, propensity-matched outcomes revealed notable differences in mortality based on the timing of admission. STEMI-CS patients admitted on weekdays experienced higher in-hospital mortality rates compared to those admitted on weekends. Conversely, NSTEMI-CS patients admitted on weekdays had lower in-hospital mortality rates than those admitted on weekends. Furthermore, STEMI-CS patients admitted on weekends were more likely to suffer an acute stroke and require pacemaker implantation compared to weekday admissions. These findings shed light on the varying characteristics and outcomes of STEMI-CS and NSTEMI-CS patients based on the timing of hospital admission, suggesting that management strategies may differ between weekdays and weekends. However, the “weekend effect” was not consistently observed across all analyses of in-hospital mortality, indicating that its influence may not uniformly apply to all patient groups or clinical outcomes. This highlights the complexity of the weekend effect and underscores the need for further investigation into how admission timing impacts clinical outcomes in acute coronary syndromes. The higher weekday mortality observed among STEMI-CS patients after matching may reflect residual confounding and unmeasured clinical severity not captured in administrative data; therefore, this association should not be interpreted as causal.

Although the propensity score-matched analysis found higher rates of in-hospital mortality among NSTEMI-CS patients admitted on weekends, prior studies have seen success in strategies to mitigate the “weekend effect.” These strategies include ensuring strict adherence to hospital protocols. For example, in 2018, Raymond et al. established standardized protocols for off-hours care in acute stroke management. They found that this increased adherence and improved outcomes for stroke management during nights and weekends [22]. Similar protocols have found success in the management of STEMI patients [23]. Another strategy for improving STEMI outcomes is the use of 24/7 in-house interventional cardiology teams to eliminate personnel shortages, which might contribute to worse outcomes during off-hours [24].

There were no statistically significant differences in other outcomes such as sudden cardiac arrest, PE, sepsis, or cardiac arrhythmias. While admission timing influenced certain complications, such as short-term mortality and stroke, it did not affect others. This discrepancy may be attributed to the critical importance of rapid intervention in acute stroke, which has been shown to improve outcomes [25,26]. Indeed, previous studies have identified a “weekend effect” in acute ischemic stroke, with patients admitted on weekends experiencing worse outcomes [27]. This phenomenon appears to be multifactorial, likely driven by reduced staffing levels and limited clinical resources during weekends [28,29].

Weekday versus weekend differences also existed in patient comorbidities. STEMI-CS patients admitted on weekdays were more likely to have prior myocardial infarctions and hypothyroidism, while NSTEMI-CS patients admitted on weekdays had a higher prevalence of smoking history. STEMI-CS patients admitted on weekdays had higher rates of prior myocardial infarctions and hypothyroidism. For NSTEMI-CS patients, those who smoked were more likely to be admitted on weekdays. One possible explanation for these differences is that patients with these comorbid conditions have already established follow-up care with their clinicians [30,31]. Therefore, these patients may be more likely to seek care earlier in the week as part of their scheduled follow-ups or due to their established relationships with healthcare professionals. Additionally, patients with chronic conditions may have more regular interactions with healthcare providers, which could lead to earlier detection when symptoms arise. This early intervention might result in these patients presenting for care during the week when outpatient appointments are more accessible.

Another difference among STEMI-CS patients was the rate of pacemaker implants. For these patients, weekend admissions were associated with higher rates of pacemaker implants. The higher rates of pacemaker implants on weekends may indicate higher rates of complications in these patients, which might be attributed to delays or postponements of critical interventions due to personnel or clinical shortages. Prior studies have found differences in timely interventions for patients presenting with STEMI. A 10-year retrospective analysis of patients presenting with acute coronary syndrome found that those seen on the weekend were less likely to receive invasive cardiac procedures such as coronary angiography, PCI, and coronary artery bypass graft [20].

Of note, NSTEMI-CS patients did not experience weekend versus weekday differences in these complications. One possible explanation is the variation in intervention rates seen for NSTEMI patients. Although early revascularization for NSTEMI-CS patients is recommended by several cardiology society guidelines, this recommendation sees variation in adherence [32,33]. For example, in 2022, Lupu et al. found that only 43.9% of high-risk NSTEMI patients received appropriate intervention in a timely fashion [34]. In contrast, patients presenting with STEMI experience substantially higher rates of timely and appropriate intervention, with studies reporting that 76% or more receive care within recommended time frames [35,36]. This difference in appropriate intervention may therefore dampen differences in the management of NSTEMI-CS patients who present on weekdays versus weekends.

Limitations

This study has several limitations inherent to its retrospective design and reliance on the NIS database. Causal relationships cannot be established, and despite propensity score matching and multivariate adjustments, residual confounding from unmeasured variables may persist. The NIS lacks granular clinical data, such as lesion complexity, symptom severity, and procedural specifics, which limits the ability to fully account for factors influencing outcomes. Additionally, the database does not capture longitudinal outcomes or allow tracking of individual patients across hospitalizations, potentially affecting the accuracy of outcome estimates. Furthermore, holidays were not accounted for in our analysis, as the NIS database does not provide specific admission dates. This may act as a potential confounder, given that holiday staffing and resource availability can differ from regular weekdays and weekends. Future studies with more granular temporal data are needed to assess this impact. Lastly, while the large sample size enhances generalizability, variations in hospital protocols, staffing, and resource availability may limit the applicability of these findings to all healthcare settings.

## Conclusions

Our study identified notable differences in clinical outcomes based on admission timing for patients with STEMI-CS and NSTEMI-CS. STEMI-CS patients admitted on weekdays experienced higher in-hospital mortality and a lower risk of acute stroke compared to weekend admissions, along with a lower incidence of pacemaker implantation. In contrast, NSTEMI-CS patients admitted on weekends had higher in-hospital mortality than those admitted on weekdays. No significant differences were observed in other outcomes, such as percutaneous coronary intervention, sudden cardiac arrest, or sepsis, for either group. These findings suggest that the timing of admission may influence mortality outcomes in cardiogenic shock, potentially reflecting variations in hospital protocols, staffing, or resource availability. Further research is needed to investigate the underlying causes of these disparities and to develop interventions aimed at ensuring consistent, high-quality care throughout the week.

## Figures and Tables

**Figure 4:**
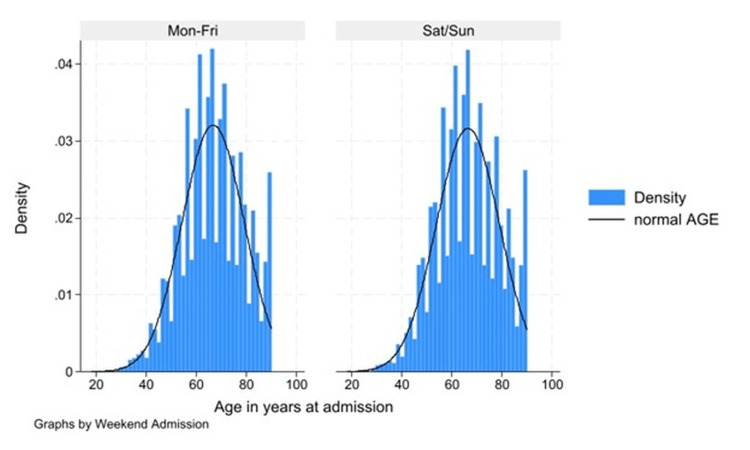
Age distribution at admission in STEMI patients on weekdays compared to weekend STEMI: ST-elevation myocardial infarction

**Figure 5:**
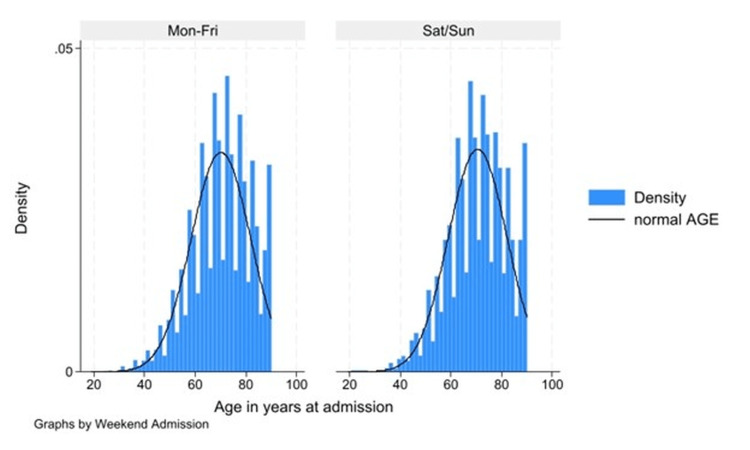
Age distribution at admission in NSTEMI patients on weekdays compared to weekend NSTEMI: non-ST-elevation myocardial infarction

**Figure 6:**
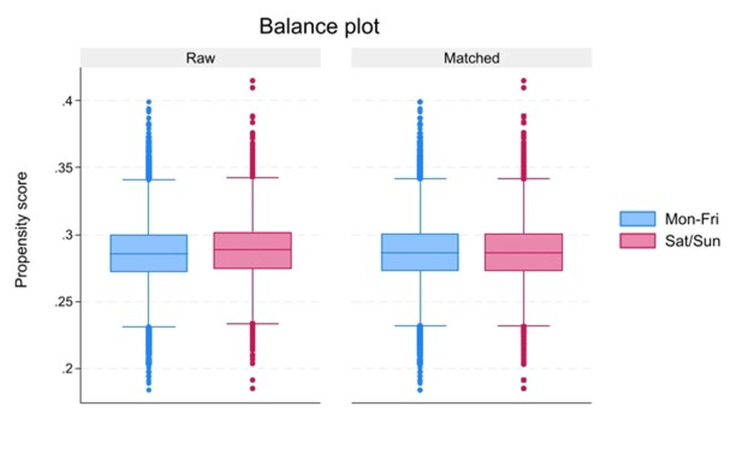
Balance plot for propensity matching in STEMI patients on weekdays compared to weekend STEMI: ST-elevation myocardial infarction

**Figure 7:**
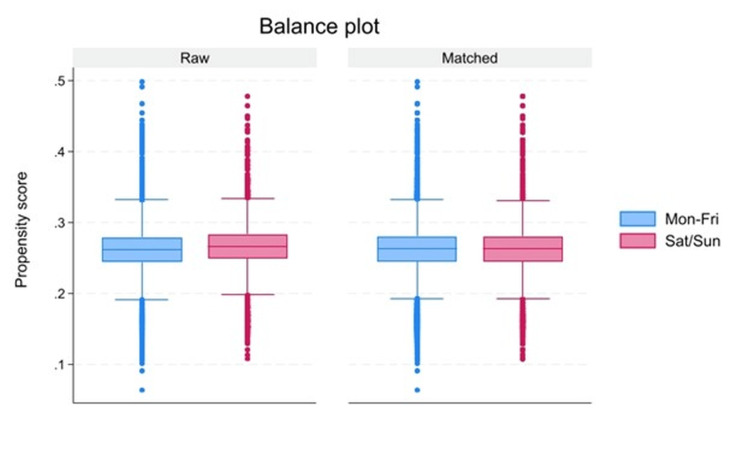
Balance plot for propensity matching in NSTEMI patients on weekdays compared to weekend NSTEMI: non-ST-elevation myocardial infarction

## Appendices

Supplementary material 1

**Table 5:** ICD-10-CM codes for included diagnoses and procedures ICD-10-CM: International Classification of Diseases, Tenth Revision, Clinical Modification; STEMI: ST-elevation myocardial infarction; NSTEMI: non-STEMI

Variable	ICD-10-CM code
STEMI	I2101, I2102, I2109, I2111, I2119, I2121, I2129, I213
NSTEMI	I214
Cardiogenic shock	R570

Supplementary material 2

**Table 6:** ICD-10-CM codes for the included outcomes ICD-10-CM: International Classification of Diseases, Tenth Revision, Clinical Modification; CPR: cardiopulmonary resuscitation

Variable	ICD-10-CM code
Acute heart failure	I5021, I5023, I5031, I5033, I5041, I5043, I50811, I50813
Acute stroke	I639, I638, I6359, I63549, I63219, I63212, I63211, I6320, I6309, I63039, I63032, I63031, I6302, I63019, I63012, I63011, I6300
Sudden cardiac arrest	I46, I97, O29, 004, 007
Sepsis	A400, A401, A410, A411, A412, A413, A414, A415, A418, A419
Pulmonary embolism	I26.XX
CPR	5A12012, 5A1221Z
Sudden cardiac arrest	I46
Pacemaker implantation	0JH604Z, 0JH605Z, 0JH606Z
Acute kidney injury	N17, N170, N171, N172, N178, N179
Defibrillator insertion	02H43KZ, 02H63KZ, 02H64KZ, 02H70KZ, 02H73KZ, 02H74KZ, 02HK3KZ, 02HK4KZ, 02HL0KZ, 02HL3KZ, 02HL4KZ, 0JH608Z, 0JH609Z, 0JH60FZ, 0JH638Z, 0JH639Z, 0JH63FZ, 0JH808Z, 0JH809Z, 0JH838Z, 0JH839Z
Percutaneous coronary intervention	027034Z, 027134Z, 027234Z, 027334Z, 02703DZ, 02713DZ, 02723DZ, 02733DZ, 02703TZ, 02713TZ, 02723TZ, 02733TZ, 0270346, 0271346, 0272346, 0273346, 02703D6, 02713D6, 02723D6, 02733D6, 02703T6, 02713T6, 02723T6, 02733T6
Cardiac arrhythmias	R001, R008, R000, I459, I456, I441, I442, I443, I47.X, I48.X, I49.X
Functional decline	Z740, Z7409, Z741, Z736

Supplementary material 3

**Table 7:** ICD-10-CM codes for cohort identification and stratification with major baseline comorbidities ICD-10-CM: International Classification of Diseases, Tenth Revision, Clinical Modification

Variables	ICD-10-CM codes
Hypertension	I10, I1150, I1151, I1152, I1158, I1159
Hyperlipidemia	E785, E7889, E7800, E781, E782, E783, E784, E784, E780, E7801, Q998, Z8639
Active smoking	F17, F172, F1720, F17200, F17201, F17203, F17208, F17209, F1721, F17210, F17211, F17213, F17218, F17219, F1722, F17220, F17221, F17223, F17228, F17229, F1729, F17290, F17291, F17293, F17298, F17299, Z87891
Obesity	E66.0, E66.1, E66.2, E66.3, E66.8, E66.9
Protein energy malnutrition	E40, E41, E42, E43, E44, E44.0, E44.1. E45, E46
Pneumonia	J12.0, J12.1, J12.2, J12.3, J12.8, J12.9, J13, J14, J15, J15.0, J15.1, J15.2, J15.3, J15.4, J15.4, J15.5, J15.5, J15.6, J15.7, J15.8, J15.8 J16, J17, J18.0, J18.1, J18.2, J18.8, J18.9
Liver disease	K70.0, K70.1, K70.2, K70.3, K70.4, K70.9, K71.0, K71.1, K71.2, K71.3, K71.4, K71.5, K71.6, K71.7, K71.8, K71.9, K72.0, K72.1, K72.9, K73.0, K73.1, K73.2, K73.8, K73.9, K74.0, K74.1, K74.2, K74.3, K74.4, K74.5, K74.6, K75.0, K75.1, K75.2, K75.3, K75.4, K75.8, K75.9, K76.0, K76.1, K76.2, K76.3, K76.4, K76.5, K76.6, K76.7, K76.8, K76.9, K77
Complicated diabetes	E10.6, E10.7, E11.6, E11.7, E13.6, E13.7, E11.21, E11.22, E11.42, E11.51
Uncomplicated diabetes	E11.9, E10.9, E13.9
Chronic kidney disease	N18.1, N18.2, N18.3, N18.30, N18.31, N18.32, N18.4. N18.5, N18.6, N18.9
Peptic ulcer disease excluding bleeding	K25.3, K26.3, K27.3, K28.3
Psychosis	F20.0, F20.1, F20.2, F20.3, F20.5, F20.8, F20.9, F22, F23, F25.0, F25.1, F25.2, F25.8, F25.9, F10.5, F11.5, F12.5, F13.5, F14.5, F15.5, F16.5, F19.5, F06.0, F06.2, F06.3, F28, F29
Depression	F32.0, F32.1, F32.2, F32.3, F32.4, F32.5, F32.8, F32.9, F33.0, F33.1, F33.2, F33.3, F33.4, F33.8, F33.9
Pulmonary disease	J449, J444, J440, J441
Hypothyroidism	E02, E30, E31, E32, E33, E34, E35, E36, E37, E38, E39
Anemia	D501 D508 D509 D500 D510 D511 D512 D513 D518 D519 D520 D528 D529 D53 D530 D531 D532 D538 D539
Valvular disease	I058, I059, I060, I061, I062, I069, I070, I071, I072, I078, I080, I081, I082, I083, I088, I089
Pulmonary circulation disorders	I2601, I2602, I2609, I2690, I2692, I2693
Renal failure	N170, N171, N172, N178, N179, N181, N182, N183, N1830, N1831, N1832, N184, N185, N186, N189, N19, N200, N201, N202, N209, N210, N211, N218, N219, N22
Fluid and electrolyte disorders	E860, E861, E869, E870, E871, E872, E873, E874, E875, E876, E8770, E8771, E8779, E878
Alcohol abuse	F1010, F1011, F10120, F10121, F10129, F10130, F10131, F10132, F10139, F1014, F10150, F10159, F10180, F10181, F10182, F10188, F1019. F1020, F1021, F10220, F101221, F10229, F10230, F10231, F10232, F10239, F1024, F10250, F10251, F10259, F1026, F1027, F10280, F10281, F10282, F10288, F1029, F10920, F10921, F10929, F10930, F10939, F1094, F10950, F10951, F10959, F1096, F1097, F10980, F10981, F10982, F10988, F1099
Drug abuse	F1110, F1011, F1110, F1111, F1210, F1211, F1310, F1311, F140, F1411, F1510, F1511, F1610, F1611, F1810, F1811, F1910, F1911
Complicated hypertension	I150, I151, I152, I158, I159, I270. I272, I2720, I2721, I2722, I2723, I2729, I87311, I87312, I87313, I87319, I87321, I87322, I87323, I87329, I87331, I87332, I87333, I87339, I87391, I87392, I87393, I87399

Supplementary material 4

**Table 8:** Proportion of missing values

Variable	Missingness
Admission month	<0.01%
Elective admission status	<0.01%
Payer type	2.5%
Race	4.9%

Supplementary material 5

**Table 9:** Matching variables used in multivariable logistics regression

Matching demographic variables
Year
Gender
Elective/non-elective
Hospital bed size
Hospital type (ownership, location, teaching status)
Hospital region
Transferred in/not transferred
Transferred out indicator
Admission day (weekend vs. weekday)
Payer type
Hypertension
Complicated hypertension
Hyperlipidemia
Obesity
Protein-energy malnutrition
Liver disease
Psychosis
Complicated diabetes
Uncomplicated diabetes
Depression
Chronic kidney disease
Smoker
Pulmonary disease
Hypothyroidism
Anemia
Valvular disease
Pulmonary circulation disorders
Renal failure
Peptic ulcer disease
Pneumonia
Fluid and electrolyte abnormalities
Alcohol abuse
Drug abuse

Supplementary material 6

Supplementary material 7

Supplementary material 8

Supplementary material 9
